# Clinical implications of thoracic duct dilatation in patients with chronic liver disease

**DOI:** 10.1097/MD.0000000000019889

**Published:** 2020-05-29

**Authors:** Seung Woon Park, Tae Hyung Kim, Soo-Youn Ham, Soon Ho Um, Hyun Gil Goh, SunHye Lee, Han Ah Lee, Sun Young Yim, Yeon Seok Seo, Hyung Joon Yim, Hyunggin An, Yu-Whan Oh

**Affiliations:** a Department of Internal Medicine, Korea University College of Medicine; b Department of Radiology, Kangbuk Samsung Hospital, Sungkyukwan University School of Medicine; c Biostatics, Korea University College of Medicine; d Department of Radiology, Korea University College of Medicine, Seoul, South Korea.

**Keywords:** ascites, esophageal and gastric varices, liver cirrhosis, thoracic duct

## Abstract

This study aimed to investigate the association between the degree of thoracic duct dilatation and the progression of chronic liver disease.

In this cross-sectional and retrospective study, 179 patients (mean age, 60.9 years; 114 men) with chronic liver disease who underwent chest CT were enrolled. Dilatation of the left distal thoracic ducts (DTD) was measured and divided into the following 3 grades according to the maximum transverse diameter: grade 0, invisible thoracic duct; grade 1, visible duct with <5-mm diameter; grade 2, diameter of ≥5 mm. Statistical analyses were conducted using the binary logistic regression model.

The proportion of grade 2 DTD was notably higher as the chronic liver disease progressed to cirrhosis. Visible DTD on chest CT was significantly related to the presence of cirrhosis (odds ratio [OR], 3.809; *P* = .027) and significant varix (OR, 3.211; *P* = .025). Grade 2 DTD was observed more frequently in patients with ascites (OR, 2.788; *P* = .039). However, 40% of patients with cirrhosis and ascites still exhibited no visible DTD while demonstrating significant amount of ascites, and their ascites were more predominant of recent onset and transient than that observed in other patients (85.7% vs 48.4%, *P* = .010 and 66.7% vs 29.0%, *P* = .009, respectively).

The degree of thoracic duct dilatation is significantly associated with progression to cirrhosis and advancement of portal hypertension. Further, insufficient lymph drainage to DTD might contribute to the development of ascites.

## 1 Introduction

Thoracic duct is the largest lymphatic channel of the body that carries chyle (comprising lymph and fats). It usually arises from the cisterna chyli and drains into the left jugular venous angle, the junctional point between the lymphatic and blood circulatory systems.^[1]^ In adults, the duct transports up to 4 L of lymph per day; however, the amount of lymph could vary depending on the pathologic condition. This is manifested as a change in the diameter of the thoracic duct.^[2]^

With the progression of chronic liver disease to cirrhosis, portal hypertension develops, not only because of increased intrahepatic resistance, especially post-sinusoidal, but also increased portal venous inflow that result from excessive dilatation of splanchnic arterioles. In addition, splanchnic hyperemia causes a reduction in effective circulating volume, which in turn activates the sympathetic nervous system and renin–angiotensin–aldosterone and vasopressin systems, aggravating portal hypertension. This vicious cycle leads to complications of cirrhosis such as gastrointestinal varix and ascites.^[3]^

In particular, ascites develops when hepatic and splanchnic lymph production exceeds the capacity of lymphatic drainage, as high portal or high sinusoidal pressure in patients with cirrhotic liver will cause a marked increase in lymph production in the liver and splanchnic organs. In fact, a markedly dilated thoracic duct has been observed in patients with far-advanced liver cirrhosis and refractory ascites or in extreme experimental conditions.^[4]^ Recently, some imaging studies^[5,6]^ showed that patients with decompensated cirrhosis showed more prominent dilatation of cisterna chyli or distal thoracic duct than those with compensated cirrhosis and non-cirrhotic liver disease. These observations reflect that the severity of the chronic liver disease generally affects the size of drainage systems of intra-abdominal lymph such as the thoracic duct.

However, the dilatation of lymphatic drainage system as a surrogate marker of progression of chronic liver disease or its clinical relevance has not been fully investigated over the entire spectrum of chronic liver disease. Further, it is uncertain whether all patients with ascites always show a definite thoracic duct dilatation. In this study, we aimed to thoroughly analyze the degree of thoracic duct dilatation in relation to the progression of chronic liver disease, and elucidate its clinical implications in the pathogenesis of ascites formation in patients with cirrhosis.

## 2 Materials and methods

### 2.1 Study design

We investigated a total of 378 patients with chronic liver disease who had undergone computed tomography (CT) from January 2014 to December 2017, through a review of their medical records. Among them, patients with hepatocellular carcinoma (n = 124), those with a history of major surgery (n = 20), those with cardiovascular disease (n = 46), and those with poor quality of CT images (n = 9) were excluded. Therefore, the remaining 179 patients were finally analyzed. The following variables were investigated: demographic information such as age and sex; platelet counts, serum albumin and bilirubin concentration, and prothrombin time (PT) international normalized ratio (INR) on the blood test; the maximum diameter of the left distal thoracic duct (DTD) on chest CT; degree of ascites, configuration of the liver, and maximum length of the spleen on abdominal CT; and the type and severity of varix, and bleeding evidence on endoscopy. The results of the blood tests performed on the date of chest CT and the image tests performed within a month before and after the date were used in the present study. The institutional review board of Korea University Anam Hospital (2018AN0093) approved this retrospective and cross-sectional study, and waived the requirement for informed consent. This study was executed in accordance with the provisions of the Declaration of Helsinki.

### 2.2 Diagnostic criteria and definitions

The presence of cirrhosis was assessed based on the liver histology, gross findings during surgery, or radiological findings showing an irregular liver margin with ascites, varices, or thrombocytopenia (<10^5^ cells/mm^3^).^[7]^

Child-Pugh grade was determined based on serum bilirubin and albumin levels, PT prolongation, and the severity of ascites and hepatic encephalopathy.^[8–10]^ Clinical ascites was defined as the ascites that required diuretics; and refractory ascites was defined as the ascites that could not be controlled even with high-dose diuretics, or was intractable owing to diuretic-induced complications, mandating repeated paracentesis.^[11]^ Recent ascites was considered as the ascites that occurred within 3 months before enrollment. Transient ascites was considered as the ascites that resolved within 3 months after enrollment for recent ascites. Hepatic encephalopathy was defined according to the West Haven Criteria for overt hepatic encephalopathy.^[12]^

The size of the varices was measured according to Beppus classification.^[13]^ Significant varix was defined as a varix size of F2 or larger, bleeding sign of the esophageal or gastric varix, or large portosystemic collateral vessels on abdomen CT.^[9,10]^ Splenomegaly was defined as the spleen measuring 12 cm or more on ultrasonography or CT.

### 2.3 Thoracic duct measurement

**Figure 1:**
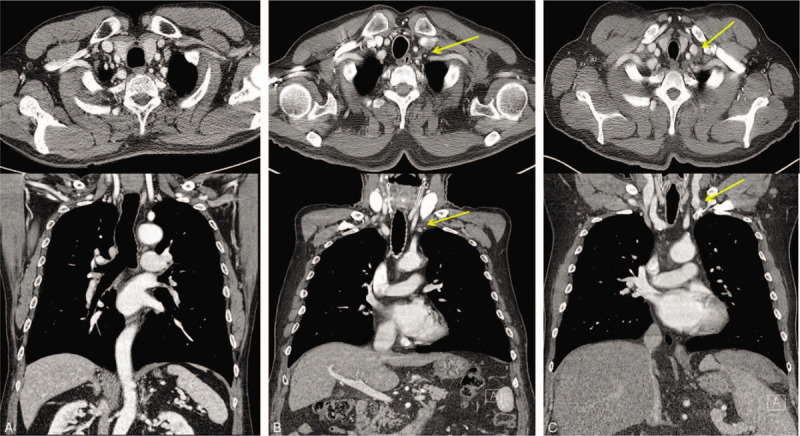
Measurement of the maximum diameter of the distal thoracic duct on the axial dimension of chest computed tomography (CT). The distal thoracic ducts noted on the axial and coronal dimensions of contrast enhanced chest CT, as indicated by yellow arrows, and the maximum diameter on axial dimension was selected. The axial and coronal images of distal thoracic duct dilatation grade 0 (A), 1 (B), and 2 (C).

Two radiologists with more than 10 years of experience reviewed CT images in a blinded manner. All chest CT scans were performed using a 128-slice dual-source CT scanner (SOMATOM Definition Flash; Siemens Healthcare, Forchheim, Germany). The images were reconstructed with 3 mm of section thickness and 3 mm of increment. The maximum diameter of the DTD was measured based on the maximum transverse dimension on chest CT, and the mean value of the measurements by 2 radiologists was considered for the study. The measurement of DTD was verified using the methods described in previous studies.^[6,14]^ Inter and intra-observer reproducibility for the measurement of DTD diameter were reliable (intra-class correlation coefficient >0.85).^[15]^ The degree of DTD dilatation was also classified into the following 3 grades: grade 0, invisible thoracic duct on chest CT; grade 1, visible thoracic duct but with a maximum diameter <5 mm; grade 2, visible thoracic duct, with a maximum diameter ≥5 mm (Fig. 1).

### 2.4 Statistics

We used SPSS version 20 (IBM, Chicago, IL, USA) and R version 3.5.2 (The R Project, Vienna, Austria) for data analyses. Continuous variables were compared using Wilcoxon Rank Sum test and Kruskal–Wallis test, while categorical variables were analyzed using the Chi-Squared test. To investigate the factors associated with thoracic duct dilatation, we conducted univariate and multivariate analyses using the binary logistic regression model. A *P* value < .05 (using two-tailed test) was considered statistically significant.

## 3 Results

### 3.1 Patient characteristics and factors associated with dilatation of DTD

The characteristics of all patients and the subgroups according to the observed DTD diameter are presented in Table 1. The median age of all patients (n = 179) was 60.1 years, and 114 of them were men. DTD was invisible (grade 0) in 109 patients, whereas it was visible in 70 patients, with the maximum diameter measured being <5 mm (grade 1) in 35 patients and a diameter of 5 mm or more (grade 2) in 35 patients.

**Table 1:**
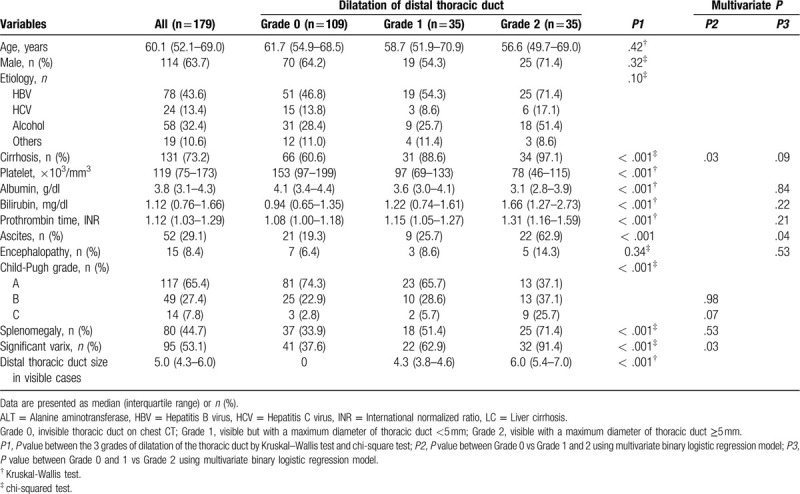
Patient characteristics and classification according to the dilatation of the distal thoracic duct.

With the increase in the grade of DTD dilatation from 0 to 2, there were more patients with concurrent cirrhosis, higher PT INR, lower serum albumin level, higher serum bilirubin level, significant varix, splenomegaly, lower platelet count, ascites, and those belonging to Child–Pugh grade C (all *P* < .001), as shown in Table 1. Univariate binary logistic regression analyses revealed that all the variables above were significantly associated with the presence of visible DTD (grade 0 vs grade 1 and 2) (all *P* < .01) or the grade 2 dilatation of DTD (grade 0 and 1 vs 2) (all *P* < .01) (Table 1).

Using multivariate binary logistic regression model, DTD visibility was shown to be independently associated with the presence of cirrhosis (OR, 3.809; 95% CI, 1.172–12.458, *P* = .027) and significant varix (OR, 3.211; 95% CI, 1.163–8.861; *P* = .025). Grade 2 dilatation of the DTD was independently associated with the presence of ascites (OR, 2.788; 95% CI, 1.051–7.393; *P* = .039).

### 3.2 DTD dilatation and other clinical findings related to disease progression

We classified patients into 4 groups based on the development of cirrhosis and the presence of complications of portal hypertension to evaluate the progression of chronic liver disease: Group 1, chronic hepatitis without cirrhosis (n *=* 48); Group 2, liver cirrhosis (LC) presenting with neither significant porto-systemic collateral vessels such as gastrointestinal varices nor ascites (n *=* 26); Group 3, LC presenting with varices or other portosystemic collateral vessels but unaccompanied by ascites (n *=* 53); and Group 4, LC accompanied by ascites (n *=* 52).

**Figure 2:**
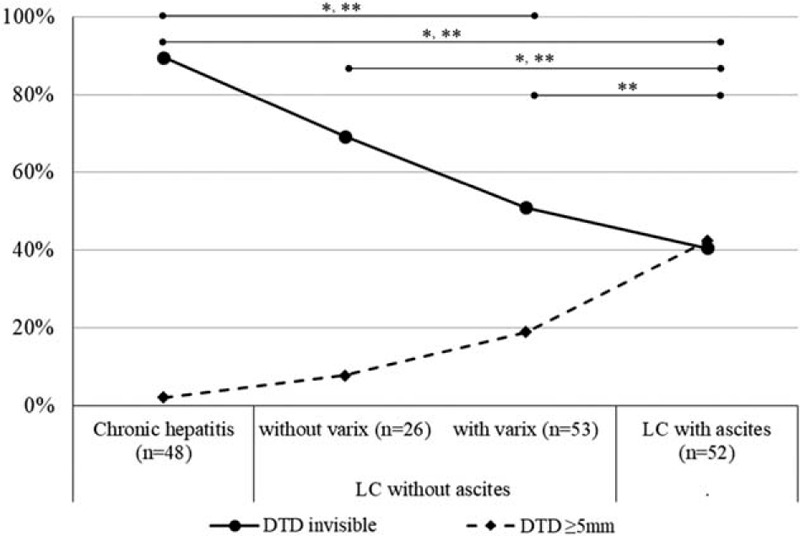
Proportion of patients with invisible distal thoracic duct and those presenting with prominent duct dilatation (>5 mm), according to the progression of chronic liver disease. The patient group with liver cirrhosis (LC) and ascites exhibited the lowest frequency of invisible (grade 0) distal thoracic duct (DTD) and the highest frequency of grade 2 dilatation of DTD, compared with the other groups (*P* < .001). ^∗^*P* < .05 for frequency of invisible DTD; ^∗∗^*P* < .05 for grade 2 dilatation of DTD.

**Figure 3:**
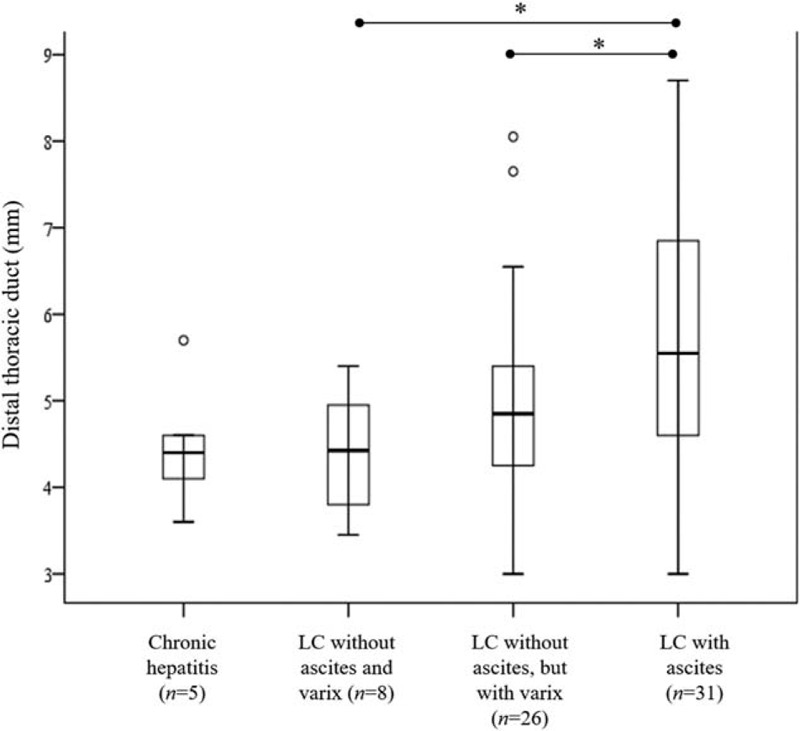
Diameter of visible left distal thoracic duct according to 4 groups divided on the basis of liver condition. The diameter of visible thoracic ducts in the liver cirrhosis (LC) with ascites group was significantly higher than that in the other groups (median [interquartile range], 5.6 [4.6–6.9] vs 4.6 [4.2–5.4] mm; *P* = .005). ^∗^*P* < .05 for comparison between 2 groups of different liver conditions.

With the progression of liver disease from group 1 to 4, the proportion of patients with invisible DTD (grade 0) decreased to 89.6%, 69.2%, 50.9%, and 40.4%, respectively. In contrast, the frequency of subjects showing grade 2 dilatation of DTD increased to 2.1%, 7.7%, 18.9%, and 42.3%, respectively (Fig. 2). Compared with other groups, group 4 patients with LC and ascites exhibited the lowest frequency of invisible (grade 0) DTD and the highest frequency with respect to grade 2 dilatation of DTD (all *P* < .001). The median diameter of the DTD in measurable cases was significantly higher in patients with LC and ascites than the diameter in patients in the other groups (*P* *=* .015) (Fig. 3).

In addition, as expected, disease progression from group 1 to 4 resulted in gradual decreases in platelet counts and serum albumin level and a steady increase in serum bilirubin level, PT, and the frequency of significant varices and portosystemic collateral vessels, splenomegaly, and Child–Pugh grade C (all *P* < .001), indicating that the liver function and hypersplenism sequentially deteriorate with the progression of chronic liver disease (Table 2).

**Table 2:**
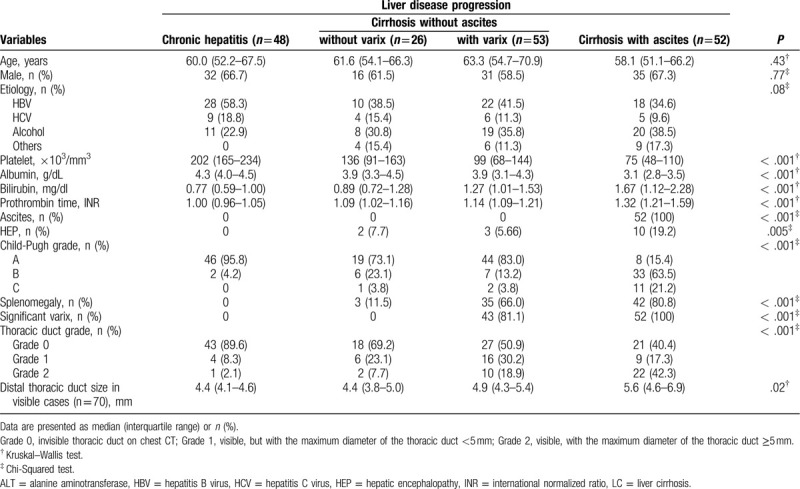
Distal thoracic duct dilatation and other clinical findings related to disease progression.

### 3.3 Clinical findings related to the degree of thoracic duct dilatation in patients with ascites

**Figure 4:**
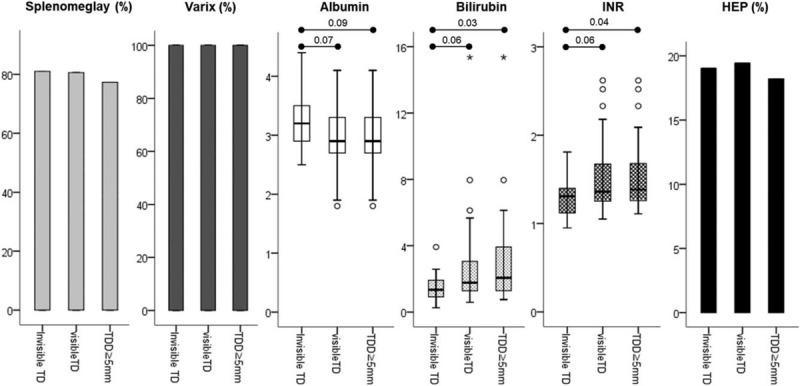
Degree of markers for severity of chronic liver disease according to thoracic duct dilatation in patients with cirrhosis and ascites. The severity of liver disease was not significantly different between patients with invisible thoracic duct and those with visible thoracic duct. It was also similar between patients with invisible thoracic duct and those with thoracic duct dilatation ≥ 5 mm, except bilirubin and INR. HEP, hepatic encephalopathy; INR, International normalized ratio; TD, thoracic duct; TDD, thoracic duct diameter. Numbers on the upper line and lower line indicate the *P*-values between groups with invisible thoracic duct and thoracic duct dilatation ≥5 mm, and groups with invisible thoracic duct and visible thoracic duct, respectively.

**Figure 5:**
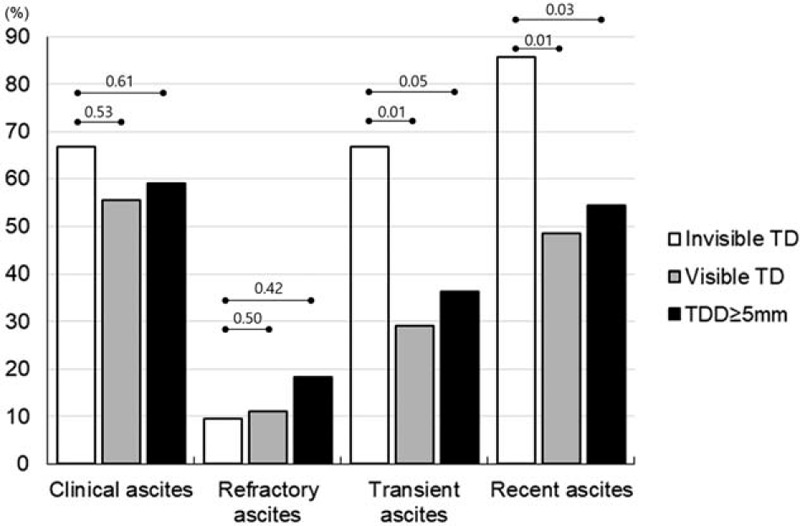
Severity and types of ascites according to the degree of DTD dilatation in patients with cirrhosis and ascites. The frequencies of clinical ascites and refractory ascites were not significantly different between the thoracic duct groups, whereas transient ascites and recent ascites were significantly more frequent in patients with invisible thoracic duct than in those with visible thoracic duct or thoracic duct dilatation ≥ 5 mm. TD, thoracic duct; TDD, thoracic duct diameter. The numbers on the upper and lower line indicate the *P*-values between groups with invisible thoracic duct and thoracic duct dilatation ≥5 mm, and groups with invisible thoracic duct and visible thoracic duct, respectively.

We compared the clinical findings related to the degree of thoracic duct dilatation among patients with cirrhosis who had ascites. There was no significant difference in the degree of ascites (i.e., comparable proportion of clinical ascites or refractory ascites), the frequency of splenomegaly, and significant varices or portosystemic collateral vessels among the 3 groups of patients showing different DTD dilatation (Table 3 and Fig. 4). In contrast, patients in whom DTD was not visible (grade 0 group) were more likely to be women (52% vs 19%, *P* = .016) and demonstrated transient ascites (66% vs 29%, *P* = .009) or recent-onset ascites (86% vs 48%, *P* = .010) significantly more often than those presenting with visible DTD (combined group of grade 1 and 2 DTD dilatation) (Fig. 5). Further, as compared to the group with grade 2 DTD dilatation only, patients in whom the DTD was not visible demonstrated significantly better liver function, as represented by their lower serum bilirubin levels (1.31 vs 2.06 mg/dl, *P* = .027) and lower PT INR values (1.31 vs 1.39, *P* = .041), as well as similar predominance of female sex and recent and transient type of ascites, although they still exhibited comparable degree of ascites with respect to its amount and refractoriness (Figs. 4 and 5).

**Table 3:**
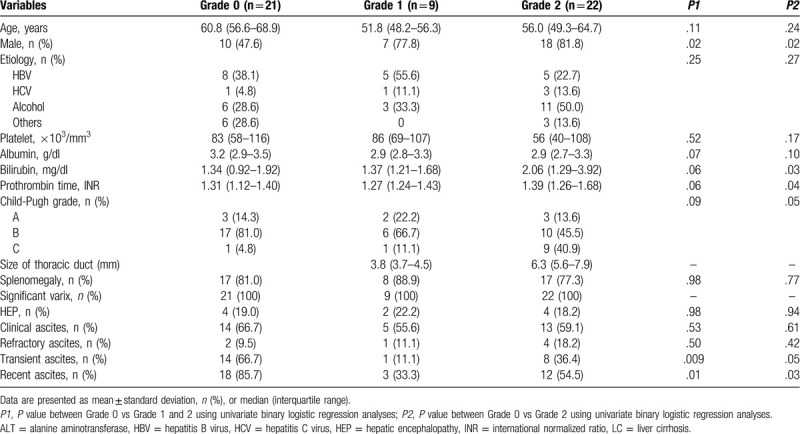
Descriptive characteristics according to the 3 grades of left distal thoracic duct dilatation in patients with cirrhosis who developed ascites.

## 4 Discussion

Chronic liver disease stiffens the liver, leading to cirrhosis, and subsequently causing portal hypertension in patients with cirrhosis. The progression of portal hypertension gradually results in appearance of typical clinical manifestations in the intra-abdominal region, including splenomegaly, esophageal and gastric varices, and finally, ascites, insinuating the presence of a considerable degree of high portal or hepatic venous pressure gradient.^[16]^

Moreover, previous researches suggested that cirrhosis-induced high hydrostatic pressure in the capillary bed of splanchnic organs or hepatic sinusoids might influence the lymph production in those organs, affecting the size of the thoracic duct as well as the drainage passage for peri-hepatic lymph, and they demonstrated that the degree of thoracic dilatation could serve as a marker for cirrhosis and its severity.^[4–6,17]^ Further, a remarkably increased lymph inflow to the thoracic duct, several times as much as that observed in normal conditions, was actually observed in patients with far-advanced cirrhosis and marked dilatation of the DTD.

However, the chronological changes of the thoracic duct in the process of progression of chronic liver disease and independent factors associated with thoracic duct dilatation observed in patients with chronic liver disease have not been sufficiently elucidated. Further, it is still uncertain whether the dilatation of the thoracic duct is an invariable phenomenon that is always observed in patients with advanced cirrhosis.

The present study clearly shows that the grade of dilatation of the DTD observed in patients with chronic liver disease is closely associated with the development of cirrhosis, validating the findings reported in previous studies. Further, it reveals that the presence of varices and ascites, rather than biochemical dysfunction, is independently correlated with the dilatation of the DTD (Table 1). This suggests that the development of portal hypertension and subsequent hemodynamic changes in the portal venous territory are mainly involved in the increase in lymphatic inflow through the thoracic duct, as has been theoretically speculated. In fact, the grade of dilatation of the DTD did not significantly differ between patients with cirrhosis but lacking the signs of portal hypertension such as gastroesophageal varices or obvious portosystemic collateral vessels and those who simply remained in the stage of chronic hepatitis (Table 2 and Fig. 1). The decreased oncotic pressure caused by hypoalbuminemia might be partially engaged in the overproduction of lymph in the splanchnic capillary bed, as suggested in univariate analysis. Of course, other causes besides portal hypertension can lead to TDD in patients with chronic liver disease. However, their potential involvement were excluded in our patient population before analysis, including iatrogenic – surgical, radiotherapy, embolization; tumor – Pancoast (left sided), primary/metastatic to posterior mediastinal compartment, lymphoma, neurogenic; idiopathic - tuberous sclerosis with chylous pleura effusions; etc.

Another important finding of the present study is that all patients with cirrhosis who had ascites exhibited obvious portosystemic collateral vessels such as varices, and they displayed more prominent dilatation of the DTD than patients with varices but without ascites (Table 2 and Fig. 1). This finding suggests that ascites basically develops in patients with cirrhosis in whom the portal hypertension has already sufficiently progressed to cause variceal development, and it just appears when the overly produced lymph in the liver and splanchnic organs distends the thoracic duct and its amount finally exceeds the limited draining capacity of the intra-abdominal lymphatic systems including the thoracic duct, as a final conduit. These results, unlike those of other studies conducted so far, exquisitely reveal the timing and mechanism of ascites formation in the process of progression of cirrhosis.

However, it should be noted that approximately 40% of patients with ascites still do not demonstrate obviously visible thoracic duct on enhanced CT images despite the presence of a significant degree of ascites. In fact, in 2 (28%) out of 7 patients with tense refractory ascites, we were unable to track the DTD on neck CT images. Although we accept the limitation of the imaging modality, this indicates that lymph inflow draining into the DTD was insufficient to distend the DTD of the majority of these patients. Two mechanisms could probably explain this phenomenon: 1 is the insufficient production of hepatic and splanchnic lymph, and the other is the insufficient transport of overly produced lymph under portal hypertension. Yet, the former mechanism seems implausible because insufficient production of lymph cannot explain the overflow of lymph resulting in ascites. Ultimately, the present study suggests that a relatively insufficient transport of the lymph produced in the liver and splanchnic organs occurs in some patients with cirrhotic ascites, and this mechanism might be responsible for the development of ascites in addition to the overproduction of lymph in these organs. This hypothesis is more supported by the fact that although patients without visible thoracic duct dilatation showed significant ascites, the type of ascites was mostly recently developed 1 or the 1 showing transient course later, which suggests that in the early phase of ascites development, the lymphatic systems of the liver and gastrointestinal tract possibly failed to catch up with the sudden overproduction of lymph in each organ under portal hypertension. In addition, compared to patients exhibiting typically dilated DTDs, these patients had more compensated liver function, also suggesting that they did not have sufficient time to adapt their intra-abdominal lymphatic system to the acutely progressing portal hypertension.

The present study has certain limitations. First, since the present study was retrospective, a selection bias might have arisen. Second, the nature of dilatation of the DTD on chest CT was not confirmed by invasive procedures, because the structures were regarded as normal. Third, we could not compare the thoracic duct dilatation and the degree of portal hypertension, because there were only a few patients whose hepatic venous pressure gradient was measured on the date of chest CT. Instead, we used manifestations of portal hypertension such as the degree of splenomegaly, varix size, and ascites. Despite these limitations, to the best of our knowledge, this study is the first to reveal the association between the dilatation of the thoracic duct and the progression of chronic liver disease and portal hypertension and suggests a new pathogenesis of ascites formation through a detailed analysis of patients with variable spectrum of chronic liver diseases and portal hypertension.

In conclusion, the thoracic duct dilates as chronic liver disease worsens, and it may be closely associated with the manifestations of portal hypertension. DTD is often invisible on CT in patients with cirrhosis who develop ascites, in particular, recent or transient type, suggesting that the insufficient lymph drainage to the DTD might contribute to the development of ascites. A further large-scale prospective study is warranted to evaluate the changes in thoracic duct dilatation and portal hypertension or liver function.

## Acknowledgments

We thank Editage (www.editage.co.kr) for the English language review.

## Author contributions

**Conceptualization:** Seung Woon Park, Soo-Youn Ham, Soon Ho Um, Yu-Whan Oh.

**Data curation:** Seung Woon Park, Hyun Gil Goh, SunHye Lee, Han Ah Lee, Sun Young Yim, Yeon Seok Seo, Hyung Joon Yim.

**Formal analysis:** Seung Woon Park, Tae Hyung Kim, Hyunggin An.

**Investigation:** Soo-Youn Ham.

**Methodology:** Soo-Youn Ham, Hyunggin An, Yu-Whan Oh.

**Project administration:** Soo-Youn Ham.

**Supervision:** Soon Ho Um.

**Visualization:** Soo-Youn Ham, Yu-Whan Oh.

**Writing – original draft:** Tae Hyung Kim.

**Writing – review & editing:** Soo-Youn Ham, Soon Ho Um.

## References

[1] Johnson OW Chick JF Chauhan NR. The thoracic duct: clinical importance, anatomic variation, imaging, and embolization. Eur Radiol 2016;26:2482–93. 26628065 10.1007/s00330-015-4112-6

[2] Phang K Bowman M Phillips A. Review of thoracic duct anatomical variations and clinical implications. Clin Anat 2014;27:637–44. 24302465 10.1002/ca.22337

[3] Piano S Tonon M Angeli P. Management of ascites and hepatorenal syndrome. Hepatol Int 2018;12:122–34. 28836115 10.1007/s12072-017-9815-0

[4] Witte MH Witte CL Dumont AE. Progress in liver disease: physiological factors involved in the causation of cirrhotic ascites. Gastroenterology 1971;61:742–50. 5117639

[5] Verma SK Mitchell DG Bergin D. Dilated cisternae chyli: a sign of uncompensated cirrhosis at MR imaging. Abdom Imaging 2009;34:211–6. 18219518 10.1007/s00261-008-9369-7

[6] Hwang SH Oh YW Ham SY. Evaluation of the left neck distal thoracic duct in cirrhosis with computed tomography. Clin Imaging 2016;40:465–9. 27133688 10.1016/j.clinimag.2016.01.005

[7] Kim TH Ku DH Um SH. How can we improve the performance of Model for End-Stage Liver Disease sodium score in patients with hepatitis B virus-related decompensated liver cirrhosis commencing antiviral treatment? J Gastroenterol Hepatol 2018;33:1641–8. 10.1111/jgh.14128 29462844

[8] Pugh RN Murray-Lyon IM Dawson JL. Transection of the oesophagus for bleeding oesophageal varices. Br J Surg 1973;60:646–9. 4541913 10.1002/bjs.1800600817

[9] Tarantino G Citro V Conca P. What are the implications of the spontaneous spleno-renal shunts in liver cirrhosis? BMC Gastroenterol 2009;9:89. 19930687 10.1186/1471-230X-9-89 PMC2785828

[10] Tarantino G Citro V Esposito P. Blood ammonia levels in liver cirrhosis: a clue for the presence of portosystemic collateral veins. BMC Gastroenterol 2009;9:21. 19292923 10.1186/1471-230X-9-21 PMC2662872

[11] Adebayo D Neong SF Wong F. Refractory ascites in liver cirrhosis. Am J Gastroenterol 2019;114:40–7. 29973706 10.1038/s41395-018-0185-6

[12] Hadjihambi A Arias N Sheikh M. Hepatic encephalopathy: a critical current review. Hepatol Int 2018;12:135–47. 28770516 10.1007/s12072-017-9812-3 PMC5830466

[13] Beppu K Inokuchi K Koyanagi N. Prediction of variceal hemorrhage by esophageal endoscopy. Gastrointest Endosc 1981;27:213–8. 6975734 10.1016/s0016-5107(81)73224-3

[14] Liu ME Branstetter BF 4th Whetstone J. Normal CT appearance of the distal thoracic duct. Am J Roentgenol 2006;187:1615–20. 17114559 10.2214/AJR.05.1173

[15] Rousson V. Assessing inter-rater reliability when the raters are fixed: Two concepts and two estimates. Biom J 2011;53:477–90. 21425184 10.1002/bimj.201000066

[16] de Franchis R. Evolving consensus in portal hypertension. Report of the Baveno IV consensus workshop on methodology of diagnosis and therapy in portal hypertension. J Hepatol 2005;43:167–76. 15925423 10.1016/j.jhep.2005.05.009

[17] Dumont AE Mulholland JH. Flow rate and composition of thoracic-duct lymph in patients with cirrhosis. N Engl J Med 1960;263:471–4. 13818600 10.1056/NEJM196009082631001

